# A review of ultrasound contrast media

**DOI:** 10.12688/f1000research.140131.1

**Published:** 2023-11-07

**Authors:** Ammar A. Oglat

**Affiliations:** 1 Department of Medical Imaging, Faculty of Applied Medical Sciences, The Hashemite University, Zarqa, 13133, Jordan., The Hashemite University, Az-Zarqa, Zarqa Governorate, 13133, Jordan

**Keywords:** contrast agents for ultrasound, Microbubbles, echocardiographic, M-mode

## Abstract

There have been numerous attempts over the past 50 years to develop efficient ultrasound contrast media (UCM) for both cardiac and noncardiac uses. In other words, the first UCM was created in the 1980s as a result of early reports from the 1960s documenting the findings of ultrasound contrast enhancement by microscopic gaseous bubbles during echocardiographic tests. A nontoxic UCM (Definity, Optison, Sonazoid, and SonoVue are some of the current commercial contrast media for echography that have shown promise in a range of on- and off-label clinical purposes) that may be injected intravenously, travels through the pulmonary, cardiac, and capillary circulations, and is stable for recirculation would be ideal. Many potential UCMs have been developed or are currently being worked on. The understanding of the physical, chemical, and biological behavior of microbubbles has greatly advanced in increase tumor vascularity, highlight sites of ischemia, and improve the ability to see vascular stenosis. Future potential, like ultrasonic molecular imaging and therapeutic uses of microbubbles, are anticipated to benefit from new inventive advancements.

## Introduction

In obstetrics, cardiology, and radiology, ultrasound imaging is a common clinical tool for the morphological examination of soft tissues. ^1^^–^^7^ As an ultrasonic wave—a longitudinal wave—travels through the body, tissue surfaces with various acoustic characteristics, such as speed of sound and density, produce reflections. The same transmitting transducer captures these scattered impulses and uses them to create an image. However, because to the size and characteristics of red blood cells, the intrinsic scattering from the blood pool is often several orders of magnitude lower than tissue at standard diagnostic frequencies (1–9 MHz). As a result, blood appears black on typical ultrasound images, making it difficult to determine the properties of blood flow. Doppler techniques can be used to measure blood velocity in bigger veins by comparing the relative motion of red blood cells to the surrounding tissue. ^8^^,^^9^ This technique is frequently used in clinical settings ( *e.g.*, obstetrics, ^10^ assessment of peripheral artery disease, ^11^ cardiology ^12^). However, this method has drawbacks when applied to areas with poor blood flow, significant tissue motion, and/or low hematocrit percentage. ^13^^–^^15^

Throughout the past few decades, ultrasound imaging’s diagnostic uses have greatly increased. The development of UCM has led to the provision of new useful physiologic and pathologic data, and the availability of perfusion imaging of cardiac or tumor tissue for everyday clinical decision-making. ^16^^,^^17^ The early 1960s saw the first reports of the ultrasonic contrast effect was studies by Joyner. Further research revealed the existence of UCM made of saline, indocyanine green, hydrogen peroxide, dextrose, and renografin. ^16^^,^^18^ UCM comprise of a suspension of small spheres of gas with a poor solubility in blood ( *e.g.*, perfluorocarbon), often ranging in size from below 10 μm in diameter. The relatively large size of UCMs guarantees that they remain strictly intravascular and function as red blood cell tracers, in contrast to contrast media employed in other modalities like (magnetic resonance imaging) MRI and computer tomography (CT). ^19^

Around 1980, achieving stability long enough for the UCM to reach the correct heart was one of the primary objectives in creating efficient UCMs. Left heart contrast was not possible until the 1990s because lung capillaries are effective filters. In 1995, contrast-enhancing substances with enhanced blood pool enhancement capabilities first surfaced. The next goal was to create bubbles that would allow for real-time imaging. In order to achieve this, air was substituted with weakly soluble gases, such as perfluorocarbons, which increased bubble endurance and allowed the development of software algorithms that could effectively distinguish UCM from tissue signals. ^20^^–^^23^

Microbubbles vibrate about their equilibrium radius in an ultrasonic field due to the compressibility of their gas cores, and they have scattering cross-sections that are many orders of magnitude higher than a solid particle of the same size. ^16^^,^^24^^,^^25^ A thin biocompatible encapsulation layer, often a phospholipid monolayer, stabilizes the bubbles by striking a balance between their ability to vibrate freely and their resistance to dissolving in-vivo during timeframes important for imaging, like half-lives of minutes. ^26^^,^^27^

Contrast echocardiography has a virtually limitless potential. Contrast echocardiography is currently the subject of extensive interest and research, as this review demonstrates. The creation of novel contrast-producing chemicals is arguably the most intriguing component of this research. It will be fascinating to watch how these different agents grow. Ideally, one or more of these novel agents will be able to cross the capillaries, allowing for peripheral venous injection-based visualization of the left side of the heart.

## Emerging technique

Over the world, contrast-enhanced ultrasound imaging is used in numerous medical and off-label applications. On multiple fronts, including the creation of novel pulse sequences and image processing techniques, the development of devices, and the creation of remote monitoring for ultrasonic therapies, this field is seeing cutting-edge breakthroughs at the same time.

### Contrast media

The only UCM that has received clinical approval is microbubbles. These bubbles have the advantage of remaining intravascular because of their size, making it possible to perform diagnostic tests that would be challenging with diffusible tracers. The use of these “conventional” UCM is being expanded, though, to include molecular-based imaging, imaging of the extravascular space, and as a platform for both imaging and therapeutic administration. ^28^^–^^32^

### Creation of the “optimal” UCM

Extensive study was done beginning in 1980 to establish contrast echocardiography as a recognized diagnostic method. ^33^ Ophir and Parker (1989) provided a summary of UCM’s application in medical imaging. ^34^ Free gas bubbles, encapsulated gas bubbles, colloidal suspensions, emulsions, and aqueous solutions were the five categories of agents that were categorized according to their physical characteristics. Producing the “perfect” contrast media that would satisfy the following requirements was still a major difficulty in those days. Such as, distribution of the substance inside the myocardial or heart chambers, which is indicative of regional blood flow; agent’s capacity to endure after an intravenous infusion during an imaging test; containing microbubbles with a diameter of less than 8 mm (smaller than red blood cells), allowing passage via the pulmonary system and the body’s smallest capillaries; good safety profile, physiological inert; and strong, regulated, and echogenic acoustic interaction.

### Synthesis of functionalized microbubbles

In 1984 (Feinstein *et al.* 1984), cavitation was used to create microbubbles after inserting the tip of a sonicator horn into a solution of human serum albumin. ^17^ This solved the problem of creating stable encapsulated microbubbles that could survive passage through the heart and the pulmonary capillary network. After a peripheral venous injection, these microbubbles could be seen in the left heart. Due to the creation of functionalized microbubbles, ^35^ or microbubbles with one or more targeted moieties inserted into the phospholipid encapsulation, ^36^ non-invasive imaging of pathophysiological events has recently been demonstrated to be viable with ultrasound. Target sites have focused on internal vasculature processes such inflammation, ^37^ angiogenesis, ^38^ and thrombus formation ^39^ since microbubbles are purely intravascular. See Table 1.

**Table 1.** Lists the most popular microbubble-based intravenous UCM at various stages of development. ^40^

Trademark name	Manufacturer	Formulations
Bisphere	Point Biomedical	Albumin/air
Echogen	Sonus Pharmaceuticals	Surfactant
Echovist	Schering AG	Galactose/air
Optison	Amersham Health Inc.	Protein-type A/perfluoropropane

The development of the first microbubbles that met the majority of the requirements for an intravenous UCM also sparked intense research by doctors, scientists, and the makers of ultrasound equipment to explain the physical phenomena and apply what they learned to therapeutic settings.

There were several technologies looked at to stabilize the microbubbles. For the purpose of lowering surface tension and stabilizing the gas core against quick dissolution, thin shells consisting of protein, polymer, or phospholipids were utilized. Unfortunately, due to the high solubility of air in water, the first-generation agents still had poor stability and relatively short circulation times. By substituting perfluorinated gases with low solubility in water, such as sulphur hexafluoride, perfluoropropane, or perfluorobutane for air during circulation, persistence during circulation was dramatically improved, resulting in sufficient persistence of the agent in the blood circulation for clinical use. ^41^

There are many ultrasound-sensitive sub-micron agents currently being researched. This research is motivated by the enhanced-permeability and retention effect, ^42^ whereby small nanometer sized particles locally extravasate from leaky blood vessels and accumulate in the perivascular space of solid tumors. Phase-shift droplets, ^43^ nanobubbles, ^44^ gas vesicles, ^45^ echogenic liposomes, ^46^ and polymeric nanoparticles ^47^ are a few of the more common examples. Although research into the physics of acoustic droplet vaporization is still ongoing, it is most probable that both intrinsic and external elements play a role in the process.

## Ultrasound imaging techniques using UCM

**Figure 1.**
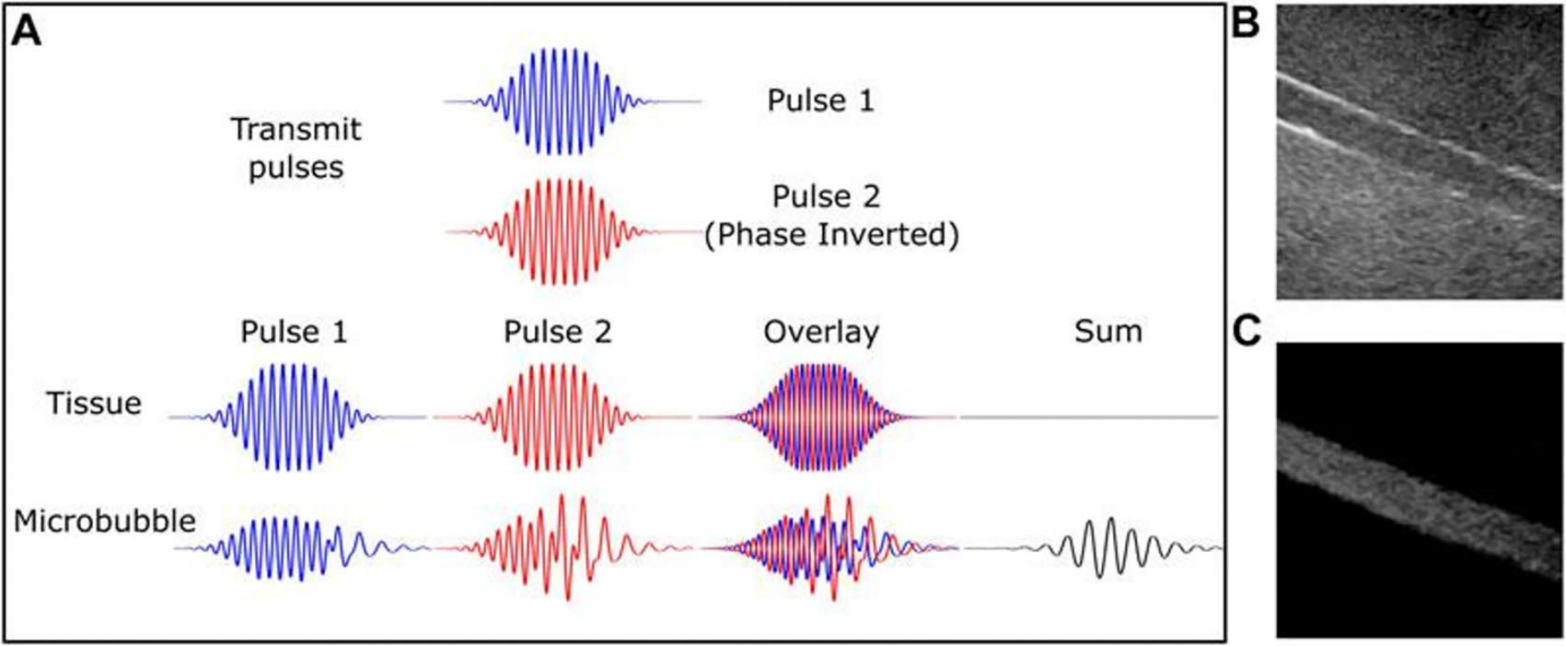
Microbubble-specific imaging sequences capture the nonlinear signal from the contrast agent while excluding tissue with linear scattering. Diagrammatic representation of the pulse inversion technique in (A). Two pulses that are 180 degrees out of phase will result in out-of-phase tissue echoes. On the other hand, this is not the case for microbubbles because of their nonlinear behavior. When an echo is produced by a microbubble, there is a significant signal, however when an echo is produced by linear tissue, the summed echo almost completely cancels out. (B) B-mode and (C) contrast-specific imaging of an 8 mm artery phantom show the enhanced vascular contrast caused by microbubble-specific imaging. This was recorded with a Philips iU22 scanner, a C5-2 probe, and the contrast medium Definity™. This number is taken from this source. Microbubble-specific imaging sequences capture nonlinear signal from contrast agent while rejecting linear scattering tissue, Yusefi, H., & Helfield, B. (2022), https://www.frontiersin.org/files/Articles/791145/fphy-10-791145-HTML/image_m/fphy-10-791145-g002.jpg, CC BY 4.0. ^28^

Vibrating microbubbles’ nonlinear nature is essential to their efficiency as an ultrasonic contrast agent. These emissions allow for the separation of bubble signals from the surrounding (about linear) tissue from those within tiny vessels. Thus, certain microbubble imaging modes were created concurrently with the advancements in UCM and as a result of a better knowledge of non-linear microbubble behavior; these are now used in the majority of clinical ultrasound systems. ^48^^–^^50^ The first methods of bubble identification were harmonic imaging, which involved gathering and filtering energy from the receive signal at the second harmonic, which is twice the driving frequency. Because the second harmonic signal produced by microbubbles is substantially greater than the second harmonic signal produced by tissue, it has a higher signal-to-noise ratio than the fundamental energy. The success of low mechanical index (MI) (0.1) contrast-specific imaging, which is primarily employed for real-time perfusion and intra-cavitary measurements, is particularly explained by the non-linear shell behavior. Furthermore, a number of diagnostic imaging procedures and/or quantification strategies are founded on the distinct and extremely sensitive attribute of microbubble destruction. ^51^ See Figure 1.

In other words, the reflection pattern from the bubble to the ultrasound signal is significantly altered by UCM. They start by greatly boosting the backscattered signal. ^23^ UCM resonate linearly in response to acoustic pressure. Acoustic pressure increases cause nonlinear vibrational patterns to manifest. ^52^ Only at higher mechanical indices (MI) do tissues create harmonic resonances, making it easy to distinguish between the signal’s tissue or UCM origin. Multiples of the natural frequencies are received using filter devices, allowing for some background (non-UCM) signal reduction. Microbubbles are disrupted by high pressure levels, which results in strong signals and signals with various properties.

## M-mode echocardiograms using UCM

Understanding the interaction between ultrasonic waves and gaseous microbubbles was made much easier by using the process utilized to explain the set of echoes first discovered on M-mode echocardiograms. The strong compressibility of the gas core appears to be particularly significant since it produces frequency-dependent volume pulsations with a clear maximum at the resonance frequency, which is inversely proportional to the size of the microbubbles. ^24^^,^^53^ However, In the following circumstances, UCM is advised by cardiologic guidelines: if the left ventricular (LV) cavum does not have two continuous segments, If the original spectrum signals are insufficient, to enhance Doppler evaluations, when periodic evaluation of the ejection fraction is necessary given the reduced variability caused by UCM, and in the case of Takotsubo myopathy, left ventricular (LV) aneurysms, and intracavitary thrombi. ^52^^,^^54^^,^^55^

## Microbubbles-based color Doppler ultrasound

Acoustic color Doppler is an imaging method that overlays color-coded maps of tissue velocity on grey-scale images of tissue anatomy. It combines anatomical information acquired from ultrasonic pulse-echo techniques with velocity information derived from ultrasonic Doppler techniques. The technique is most frequently used to visualize blood flow through the heart, arteries, and veins, but it can also be used to visualize the movements of solid tissues like the walls of the heart. vectors. Almost all commercial ultrasound equipment now provides color Doppler imaging, which has been proven to be very useful in determining blood flow in a variety of clinical circumstances. Although the technique for getting velocity information is quite similar to the technique for getting anatomical information, there are a number of reasons why it is technically more difficult. It also has a few flaws, the biggest of which is that, in conventional systems, the velocities measured and subsequently displayed are the components of the flow velocity directly towards or away from the transducer, whereas the method’s ideal output would provide data on the magnitude and direction of the three-dimensional flow vectors. ^56^^–^^61^

In conjunction with color or power Doppler, stimulated acoustic emission is employed in high mechanical index (MI) imaging. A high MI ultrasound impulse is used to deflate the microbubbles, and the signal that is received is a complicated mixture of ultrasound waves that causes a Doppler shift. ^62^ It is especially helpful when a UCM with tissue specificity, is in its late stages. ^20^ Color Doppler imaging has been shown that despite the poor spatial resolution, real-time imaging was possible due to the tiny size of the picture window. ^63^

Recently, the use of ultrasound color Doppler has been shown to monitor bubbles during ultrasound therapy. An active ultrasound imaging method called color Doppler uses the phase shifts between the echoes of imaging pulses to measure velocities. In the aforementioned investigation, an increase in the color Doppler signal was connected with the formation of cavitation bubbles on their own under a high-pressure ultrasonic beam. The rise was utilized to evaluate tissue fractionation and was related to the mobility of the surrounding tissue brought on by cavitation within the focused location. Although this method is helpful for high-pressure therapy, it does not reveal how net bubbles flow through the field. ^64^^,^^65^

## Modern commercial UCM

All currently marketed UCM are made up of an inert gas enclosed in a shell. The gas determines solubility and most of the acoustic qualities of the bubbles, while the shell mostly affects the viscoelastic properties, such as stability and durability. ^66^ Perfluorocarbon bubbles, which range in size almost 10 m and are real blood pool agents, allow transit through the pulmonary vascular system, which is necessary for entry to the systemic circulation. ^52^ Soft shell materials have better nonlinear oscillations and are made of phospholipids or other surfactants. ^67^ There are also protein-shelled microbubbles that contain an albumin shell around perfluoropropane gas.

## Conclusion

We outlined the basics of contrast agent microbubble vibration and how it is used for common contrast-imaging applications in this paper. Throughout the past five decades, UCM imaging has made significant advancements. The main clinical focus in the past was echocardiography, with myocardial perfusion being referred to as the “holy grail” and being seen as a significant market for contrast echo. The main reason for echocardiography was LV opacification, which improves LV endocardial border delineation. UCM imaging is a safe and effective method for many clinical applications, and its use is growing. Due to increased clinical awareness of ultrasound’s advantages as well as collaborative research projects between physicists, chemists, engineers, and clinicians on the study of microbubble behavior, signal processing methods, contrast agent synthesis, and device development, this imaging technology has achieved tremendous success to date.

## Data Availability

No data are associated with this article.

## References

[1] Oglat AA : A review of medical doppler ultrasonography of blood flow in general and especially in common carotid artery. *J Med Ultrasound.* 2018;26(1):3–13. 10.4103/JMU.JMU_11_17 30065507 PMC6029191

[2] Oglat AA : A new scatter particle and mixture fluid for preparing blood mimicking fluid for wall-less flow phantom. *J Med Ultrasound.* 2018;26(3):134–142. 10.4103/JMU.JMU_7_18 30283199 PMC6159322

[3] Ammar AO : Characterization and construction of a robust and elastic wall-less flow phantom for high pressure flow rate using Doppler ultrasound applications. *Nat Eng Sci.* 2018;3(3):359–377. 10.28978/nesciences.468972

[4] Oglat AA : *Artifacts in diagnostic ultrasonography.* Los Angeles, CA: SAGE Publications Sage CA;2020.

[5] Shalbi SM : A brief review for common doppler ultrasound flow phantoms. *J Med Ultrasound.* 2020;28(3):138–142. 10.4103/JMU.JMU_96_19 33282656 PMC7709522

[6] Dakok KK : A review of carotid artery phantoms for doppler ultrasound applications. *J Med Ultrasound.* 2021;29(3):157–166. 10.4103/JMU.JMU_164_20 34729323 PMC8515632

[7] Athamnah SI : Diagnostice breast elastography estimation from doppler imaging using central difference (CD) and least-squares (LS) algorithms. *Biomedical Signal Processing and Control.* 2021;68:102667. 10.1016/j.bspc.2021.102667

[8] Oglat AA Dheyab MA : Performance evaluation of ultrasonic imaging system (Part I). *J Med Ultrasound.* 2021;29(4):258–263. 10.4103/JMU.JMU_166_20 35127405 PMC8772471

[9] Oglat AAJB : Performance Evaluation of an Ultrasonic Imaging System Using Tissue-Mimicking Phantoms for Quality Assurance. *Biomimetics.* 2022;7(3):130. 10.3390/biomimetics7030130 36134934 PMC9496229

[10] Mone F : The clinical application of Doppler ultrasound in obstetrics. *Obstet Gynaecol.* 2015;17(1):13–19. 10.1111/tog.12152

[11] Grant EG : Carotid artery stenosis: gray-scale and Doppler US diagnosis—Society of Radiologists in Ultrasound Consensus Conference. *Radiology.* 2003;229(2):340–346. 10.1148/radiol.2292030516 14500855

[12] Quiñones MA : Recommendations for quantification of Doppler echocardiography: a report from the Doppler Quantification Task Force of the Nomenclature and Standards Committee of the American Society of Echocardiography. *J Am Soc Echocardiogr.* 2002;15(2):167–184. 10.1067/mje.2002.120202 11836492

[13] Cobbold RS : *Foundations of biomedical ultrasound.* Oxford University Press;2006.

[14] Jensen JA : *Estimation of blood velocities using ultrasound: a signal processing approach.* Cambridge University Press;1996.

[15] Szabo TL : *Diagnostic ultrasound imaging: inside out.* Academic Press;2004.

[16] Gramiak R Shah PM : Echocardiography of the aortic root. *Investig Radiol.* 1968;3(5):356–366. 10.1097/00004424-196809000-00011 5688346

[17] Feinstein SB : Microbubble dynamics visualized in the intact capillary circulation. *J Am Coll Cardiol.* 1984;4(3):595–600. 10.1016/S0735-1097(84)80107-2 6470341

[18] Forsberg F : Effect of filling gases on the backscatter from contrast microbubbles: theory and in vivo measurements. *Ultrasound Med Biol.* 1999;25(8):1203–1211. 10.1016/S0301-5629(99)00079-4 10576263

[19] Becher H Burns PN : *Handbook of contrast echocardiography: Left ventricular function and myocardial perfusion.* Springer Science & Business Media;2012.

[20] Cosgrove D : Ultrasound contrast agents: an overview. *Eur J Radiol.* 2006;60(3):324–330. 10.1016/j.ejrad.2006.06.022 16938418

[21] Goldberg BB : Ultrasound contrast agents: a review. *Ultrasound Med Biol.* 1994;20(4):319–333. 10.1016/0301-5629(94)90001-9 8085289

[22] Ignee A : Ultrasound contrast agents. *Endosc Ultrasound.* 2016;5(6):355–362. 10.4103/2303-9027.193594 27824024 PMC5206822

[23] Calliada F : Ultrasound contrast agents: basic principles. *Eur J Radiol.* 1998;27:S157–S160. 10.1016/S0720-048X(98)00057-6 9652516

[24] Medwin HJU : Counting bubbles acoustically: a review. *Ultrasonics.* 1977;15(1):7–13. 10.1016/0041-624X(77)90005-1

[25] Covella M : Echocardiographic aortic root dilatation in hypertensive patients: a systematic review and meta-analysis. *J Hypertens.* 2014;32(10):1928–1935. 10.1097/HJH.0000000000000286 24979304

[26] Unger EC : Therapeutic applications of lipid-coated microbubbles. *Adv Drug Deliv Rev.* 2004;56(9):1291–1314. 10.1016/j.addr.2003.12.006 15109770

[27] Garg S Thomas A Borden M : *The Effect of Lipid Monolayer In-Plane Rigidity on.* Vivo;2013. 10.1016/j.biomaterials.2013.05.053 PMC3762333 23787108

[28] Yusefi H Helfield B : Ultrasound contrast imaging: Fundamentals and emerging technology. *Front Phys.* 2022;10:100. 10.3389/fphy.2022.791145

[29] Frinking PJ : Ultrasound contrast imaging: current and new potential methods. *Ultrasound Med Biol.* 2000;26(6):965–975. 10.1016/S0301-5629(00)00229-5 10996696

[30] Averkiou M : Ultrasound contrast imaging research. *Ultrasound Q.* 2003;19(1):27–37. 10.1097/00013644-200303000-00004 12970614

[31] Correas J-M : Ultrasound contrast agents: properties, principles of action, tolerance, and artifacts. *Eur Radiol.* 2001;11:1316–1328. 10.1007/s003300100940 11519538

[32] Sofuni A : Differential diagnosis of pancreatic tumors using ultrasound contrast imaging. *J Gastroenterol.* 2005;40:518–525. 10.1007/s00535-005-1578-z 15942718

[33] Senior R : Clinical practice of contrast echocardiography: recommendation by the European Association of Cardiovascular Imaging (EACVI) 2017. *Eur Heart J Cardiovasc Imaging.* 2017;18(11):1205–1205af. 10.1093/ehjci/jex182 28950366

[34] Ophir J Parker KJ : Contrast agents in diagnostic ultrasound. *Ultrasound Med Biol.* 1989;15(4):319–333. 10.1016/0301-5629(89)90044-6 2669297

[35] Lindner JR : Microbubbles in medical imaging: current applications and future directions. *Nat Rev Drug Discov.* 2004;3(6):527–533. 10.1038/nrd1417 15173842

[36] Langeveld SA Meijlink B Kooiman K : Phospholipid-coated targeted microbubbles for ultrasound molecular imaging and therapy. *Curr Opin Chem Biol.* 2021;63:171–179. 10.1016/j.cbpa.2021.04.013 34102582

[37] Wang Y : Clopidogrel with aspirin in acute minor stroke or transient ischemic attack (CHANCE) trial: one-year outcomes. *Circulation.* 2015;132(1):40–46. 10.1161/CIRCULATIONAHA.114.014791 25957224

[38] Willmann JK : Targeted contrast-enhanced ultrasound imaging of tumor angiogenesis with contrast microbubbles conjugated to integrin-binding knottin peptides. *J Nucl Med.* 2010;51(3):433–440. 10.2967/jnumed.109.068007 20150258 PMC4111897

[39] Hamilton AJ : Intravascular ultrasound molecular imaging of atheroma components in vivo. *J Am Coll Cardiol.* 2004;43(3):453–460. 10.1016/j.jacc.2003.07.048 15013130

[40] Frinking P : Three decades of ultrasound contrast agents: a review of the past, present and future improvements. *Ultrasound Med Biol.* 2020;46(4):892–908. 10.1016/j.ultrasmedbio.2019.12.008 31941587

[41] Schneider M : BR1: a new ultrasonographic contrast agent based on sulfur hexafluoride-filled microbubbles. *Investig Radiol.* 1995;30(8):451–457. 10.1097/00004424-199508000-00001 8557510

[42] Matsumura Y Maeda H : A new concept for macromolecular therapeutics in cancer chemotherapy: mechanism of tumoritropic accumulation of proteins and the antitumor agent smancs. *Cancer Res.* 1986;46(12_Part_1):6387–6392. 2946403

[43] Sheeran PS Dayton PA : Phase-change contrast agents for imaging and therapy. *Curr Pharm Des.* 2012;18(15):2152–2165. 10.2174/138161212800099883 22352770 PMC5045864

[44] Helfield B Zou Y Matsuura N : Acoustically-stimulated nanobubbles: opportunities in medical ultrasound imaging and therapy. *Front Phys.* 2021;9:654374. 10.3389/fphy.2021.654374

[45] Shapiro MG : Biogenic gas nanostructures as ultrasonic molecular reporters. *Nat Nanotechnol.* 2014;9(4):311–316. 10.1038/nnano.2014.32 24633522 PMC4023545

[46] Kopechek JA : Acoustic characterization of echogenic liposomes: Frequency-dependent attenuation and backscatter. *J Acoust Soc Am.* 2011;130(5):3472–3481. 10.1121/1.3626124 22088022 PMC3248067

[47] Kwan JJ : Ultrasound-propelled nanocups for drug delivery. *Small.* 2015;11(39):5305–5314. 10.1002/smll.201501322 26296985 PMC4660885

[48] Burns PJCR : Harmonic imaging: principles and preliminary results. *Clin Radiol.* 1996;51:50–55. 8605774

[49] Wei K : Quantification of myocardial blood flow with ultrasound-induced destruction of microbubbles administered as a constant venous infusion. *Circulation.* 1998;97(5):473–483. 10.1161/01.CIR.97.5.473 9490243

[50] Averkiou MA : Imaging methods for ultrasound contrast agents. *Ultrasound Med Biol.* 2020;46(3):498–517. 10.1016/j.ultrasmedbio.2019.11.004 31813583

[51] Albrecht T : Improved detection of hepatic metastases with pulse-inversion US during the liver-specific phase of SHU 508A: multicenter study. *Radiology.* 2003;227(2):361–370. 10.1148/radiol.2272011833 12649417

[52] Appis AW : Update on the safety and efficacy of commercial ultrasound contrast agents in cardiac applications. *Echo Res Pract.* 2015;2(2):R55–R62. 10.1530/ERP-15-0018 26693339 PMC4676450

[53] Hu Y-Z : Ultrasound microbubble contrast agents: application to therapy for peripheral vascular disease. *Adv Ther.* 2009;26:425–434. 10.1007/s12325-009-0020-y 19381521

[54] Albrecht T : Comparison of bolus and infusion of the ultrasound contrast media levovist for color doppler ultrasound of renal arteries. *Rofo.* 2000;172(10):824–829. 10.1055/s-2000-7892 11111294

[55] Porter TR : Guidelines for the cardiac sonographer in the performance of contrast echocardiography: a focused update from the American Society of Echocardiography. *J Am Soc Echocardiogr.* 2014;27(8):797–810. 10.1016/j.echo.2014.05.011 25085408

[56] Arning C : Revision of DEGUM ultrasound criteria for grading internal carotid artery stenoses and transfer to NASCET measurement. *Ultraschall Med.* 2010;31(3):251–257. 10.1055/s-0029-1245336 20414854

[57] Udesen J : Examples of in vivo blood vector velocity estimation. *Ultrasound Med Biol.* 2007;33(4):541–548. 10.1016/j.ultrasmedbio.2006.10.014 17346874

[58] Thalhammer C : Colour-coded duplex sonography after renal transplantation. *Ultraschall Med.* 2007;28(1):6–27. quiz 25. 10.1055/s-2007-962859 17304409

[59] Parikh S Shah R Kapoor P : Portal vein thrombosis. *Am J Med.* 2010;123(2):111–119. 10.1016/j.amjmed.2009.05.023 20103016

[60] Baik SKJLI : Haemodynamic evaluation by Doppler ultrasonography in patients with portal hypertension: a review. *Liver Int.* 2010;30(10):1403–1413. 10.1111/j.1478-3231.2010.02326.x 20731772

[61] Whittingham TA : Medical diagnostic applications and sources. *Prog Biophys Mol Biol.* 2007;93(1-3):84–110. 10.1016/j.pbiomolbio.2006.07.004 16949652

[62] Albrecht T : Stimulated acoustic emissions with the ultrasound contrast medium levovist: a clinically useful contrast effect with liver-specific properties. *Rofo.* 2000;172(1):61–67. 10.1055/s-2000-11101 10719465

[63] Dietrich CF : Improved differentiation of pancreatic tumors using contrast-enhanced endoscopic ultrasound. *Clin Gastroenterol Hepatol.* 2008;6(5):590–597.e1. 10.1016/j.cgh.2008.02.030 18455699

[64] Zhang X : Real-time feedback of histotripsy thrombolysis using bubble-induced color Doppler. *Ultrasound Med Biol.* 2015;41(5):1386–1401. 10.1016/j.ultrasmedbio.2014.12.006 25623821 PMC4398659

[65] Evans DH Jensen JA Nielsen MB : Ultrasonic colour Doppler imaging. *Interface Focus.* 2011;1(4):490–502. 10.1098/rsfs.2011.0017 22866227 PMC3262272

[66] Paefgen V Doleschel D Kiessling F : Evolution of contrast agents for ultrasound imaging and ultrasound-mediated drug delivery. *Front Pharmacol.* 2015;6:197. 10.3389/fphar.2015.00197 26441654 PMC4584939

[67] Leong-Poi H : Influence of microbubble shell properties on ultrasound signal: Implications for low-power perfusion imaging. *J Am Soc Echocardiogr.* 2002;15(10):1269–1276. 10.1067/mje.2002.124516 12411916

