# Supplementary material for: A review of ultrasound contrast media
Source: F1000Res. 2024 Jun 4;12:1444. Originally published 2023 Nov 7. [Version 3] doi: 10.12688/f1000research.140131.3 (PMC11137482; doi:10.12688/f1000research.140131.3)
Supplement: Peer review history

# Reviewer report: Reviewer response for version 2

**DOI:** 10.5256/f1000research.163423.r254669

**Published:** 2024-05-29

**Authors:** Haytham Alewaidat

**Affiliations:** 1 Faculty of Applied Medical Sciences, Jordan University of Science and Technology, Irbid, Jordan

This review shows An ideal contrast medium for echography should be nontoxic, capable of being injected intravenously, able to travel through pulmonary, cardiac, and capillary circulations, and stable for recirculation

**The comments are below:**

 The author mentioned each detail about the contrast media used in ultrasound, such as the composition, applications, stability, currently commercially available ultrasound contrast agents, and ultrasound contrast agent imaging.

 Examples of current commercial contrast media include Definity, Optison, Sonazoid, and SonoVue, which have demonstrated potential for various clinical purposes, both on- and off-label. 

 Please check the grammatical and typo errors in all manuscript sections.  

 The contents of this review reflects an excellent scientific subject.

 Please note that the conclusion part requires a small correction.

Is the review written in accessible language?

Yes

Are all factual statements correct and adequately supported by citations?

Yes

Are the conclusions drawn appropriate in the context of the current research literature?

Yes

Is the topic of the review discussed comprehensively in the context of the current literature?

Yes

Reviewer Expertise:

Medical Imaging

I confirm that I have read this submission and believe that I have an appropriate level of expertise to confirm that it is of an acceptable scientific standard.

# Reviewer report: Reviewer response for version 2

**DOI:** 10.5256/f1000research.163423.r254568

**Published:** 2024-03-27

**Authors:** Hamad Yahia Abu Mhanna

**Affiliations:** 1 Universiti Sains Malaysia, Minden Heights, Pulau Pinang, Malaysia

**I agree **with all modifications made by the authors

Is the review written in accessible language?

Yes

Are all factual statements correct and adequately supported by citations?

Yes

Are the conclusions drawn appropriate in the context of the current research literature?

Yes

Is the topic of the review discussed comprehensively in the context of the current literature?

Yes

Reviewer Expertise:

Medical Imaging

I confirm that I have read this submission and believe that I have an appropriate level of expertise to confirm that it is of an acceptable scientific standard.

# Reviewer report: Reviewer response for version 1

**DOI:** 10.5256/f1000research.153460.r248551

**Published:** 2024-03-05

**Authors:** Hamad Yahia Abu Mhanna

**Affiliations:** 1 Universiti Sains Malaysia, Minden Heights, Pulau Pinang, Malaysia

**Minor comments:**

  

1. The title is accurately representing the content of the article.
2. Kindly make the contribution of this review clearer in the **abstract** section.
3. In the **abstract** section, please rephrase most of the sentences using academic language.
4. The **introduction** part is written in a thorough and complete manner. However, in the second paragraph, this is a run on sentence and hard to follow. “Throughout the past few decades, ultrasound imaging’s diagnostic uses have greatly increased. The development of UCM has led to the provision of new useful physiologic and pathologic data, and the availability of perfusion imaging of cardiac or tumor tissue for everyday clinical decision-making.”  Recommend breaking it up for clarity.
5. The **caption** of **Figure** (1) is too long and make the reader confused. Please rewrite it again in a brief way.
6. Some of grammatical errors in the **conclusion** section need correction.
7. Number of references is enough for this manuscript. Good
8. Make sure that all **sentences** are linked together and all **values** in this study are true.

Is the review written in accessible language?

Yes

Are all factual statements correct and adequately supported by citations?

Yes

Are the conclusions drawn appropriate in the context of the current research literature?

Yes

Is the topic of the review discussed comprehensively in the context of the current literature?

Yes

Reviewer Expertise:

Medical Imaging

I confirm that I have read this submission and believe that I have an appropriate level of expertise to confirm that it is of an acceptable scientific standard.

# Response

**Published:** 2024-03-07

**Authors:** Ammar A. Oglat

**Affiliations:** The Hashemite University, Az-Zarqa, Zarqa Governorate, Jordan

**Responses to Reviewer’s Comments**

**Title:  A review of ultrasound contrast media**

**Dear Editor-in-Chief,**

 The author sincerely appreciates the reviewers’ technical comments and the useful suggestions offered to us to revise our manuscript. My response to reviewers’ comments point by point are listed hereafter.

**Comments from Reviewer #1: **

1. The title is accurately representing the content of the article.

Answer: Esteemed reviewer, Thank you for your helpful comments. Yes, I tried to make the title comprehensive to cover all the manuscript content.

1. Kindly make the contribution of this review clearer in the abstract section.

Answer: The mentioned comment is corrected. The whole abstract is modified and the contribution of this review is simplify and clarify.

1. In the abstract section, please rephrase most of the sentences using academic language.

Answer: The abstract section is modified and rephrased to be in a good scientific manner.

  

1.  The introduction part is written in a thorough and complete manner. However, in the second paragraph, this is a run on sentence and hard to follow. “Throughout the past few decades, ultrasound imaging’s diagnostic uses have greatly increased. The development of UCM has led to the provision of new useful physiologic and pathologic data, and the availability of perfusion imaging of cardiac or tumor tissue for everyday clinical decision-making.”  Recommend breaking it up for clarity.

Answer: Noted and done.

  

1. The caption of Figure (1) is too long and make the reader confused. Please rewrite it again in a brief way.

Answer: Yes. The caption is modified and shortened.

  

1. Some of grammatical errors in the conclusion section need correction.

Answer: Ok. Noted and the conclusion is edited.

  

1. Number of references is enough for this manuscript. Good

Answer: Thank you very much for your valuable comments and scientific support.  

  

1. Make sure that all sentences are linked together and all values in this study are true.

Answer: I made a double check if all values in the manuscript again and there is no any error or mistake.

 Dear Respected reviewer. Thank you very much for your valuable comments which enhance the manuscript with scientific information. Appreciate
